# Targeted screening and identification of chlorhexidine as a pro-myogenic circadian clock activator

**DOI:** 10.1186/s13287-023-03424-2

**Published:** 2023-07-31

**Authors:** Tali Kiperman, Weini Li, Xuekai Xiong, Hongzhi Li, David Horne, Ke Ma

**Affiliations:** 1 Department of Diabetes Complications and Metabolism, Arthur Riggs Diabetes and Metabolism Research Institute, Beckman Research Institute of City of Hope, Duarte, CA 91010 USA; 2 Department of Molecular Medicine, Beckman Research Institute of City of Hope, Duarte, CA 91010 USA

**Keywords:** Muscle stem cell, Myogenic progenitor, Myogenesis, Myoblast differentiation, Circadian clock, Small molecule, Drug screening

## Abstract

**Background:**

The circadian clock is an evolutionarily conserved mechanism that exerts pervasive temporal control in stem cell behavior. This time-keeping machinery is required for orchestrating myogenic progenitor properties in regenerative myogenesis that ameliorates muscular dystrophy. Here we report a screening platform to discover circadian clock modulators that promote myogenesis and identify chlorhexidine (CHX) as a clock-activating molecule with pro-myogenic activities.

**Methods:**

A high-throughput molecular docking pipeline was applied to identify compounds with a structural fit for a hydrophobic pocket within the key circadian transcription factor protein, Circadian Locomotor Output Cycles Kaput (CLOCK). These identified molecules were further screened for clock-modulatory activities and functional validations for pro-myogenic properties.

**Results:**

CHX was identified as a clock activator that promotes distinct aspects of myogenesis. CHX activated circadian clock that reduced cycling period length and augmented amplitude. This action was mediated by the targeted CLOCK structure via augmented interaction with heterodimer partner Bmal1, leading to enhanced CLOCK/Bmal1-controlled transcription with upregulation of core clock genes. Consistent with its clock-activating function, CHX displayed robust effects on stimulating myogenic differentiation in a clock-dependent manner. In addition, CHX augmented the proliferative and migratory activities of myoblasts.

**Conclusion:**

Our findings demonstrate the feasibility of a screening platform to discover clock modulators with myogenic regulatory activities. Discovery of CHX as a pro-myogenic molecule could be applicable to promote regenerative capacities in ameliorating dystrophic or degenerative muscle diseases.

**Supplementary Information:**

The online version contains supplementary material available at 10.1186/s13287-023-03424-2.

## Introduction

The circadian clock, driven by a transcriptional feed-back loop coupled with translational and posttranslational regulations, generates the ∼24-h rhythm in physiology and behavior [1, 2]. This evolutionarily conserved time-keeping mechanism imparts pervasive temporal control in diverse biological processes, including metabolic regulation, cell cycle and stem cell behavior [3–8]. Disruption of circadian clock regulation, increasingly prevalent in a modern lifestyle, leads to the development of metabolic disorders [9, 10], a myriad of cancers [11], and dysregulation of tissue remodeling [6–8]. Therapeutic targeting of circadian clock and its biological output pathways may have applications in metabolic disorders, cancer treatment and prevention, and dystrophic muscle diseases [6, 11–16]. To date, small molecule compounds to target the clock for disease applications have yet to be discovered, particularly for diseases involving stem cells in tissue remodeling processes. This spurred the current interests to identify clock-modulating molecules that may have therapeutic potentials for drug development [17–19].

The molecular machinery that drives circadian rhythm, the molecular clock, is composed of a transcriptional/translational negative feed-back circuitry [2]. The key transcription activators, CLOCK (Circadian Locomotor Output Cycles Kaput) and Brain and Muscle Arnt-like 1 (Bmal1), heterodimerize via the Per-Arnt-Sim (PAS) domain to form a transcription complex that drives clock gene transcription [20]. Clock repressor proteins, Cryptochrome (Cry1 and 2) and Periods (Per1-3), are direct transcription targets of CLOCK/Bmal1, thereby constituting a negative transcriptional feed-back arm by inhibiting CLOCK/Bmal1 activity. CLOCK/Bmal1 also activates nuclear receptors, Rev-erbα and RORα, to exert positive and negative regulations via RORE response element to generate Bmal1 transcriptional oscillation, a mechanism that re-enforces the robustness of the clock [21, 22]. Additional post-transcriptional and posttranslational regulatory controls are involved to complete the molecular clock circuit that drives the 24-h oscillations in gene expression, physiology and behavior [1]. Recently, various components of the molecular clock circuit have been targeted for pharmacological modulations. However, the small molecule clock modulators discovered to date target negative regulators such as Rev-erbα and Cry [12, 13, 23–25]. Molecules that directly modulate the key driver of the molecular clock, namely CLOCK/Bmal1-mediated transcriptional activation, remain to be identified.

Accumulating studies indicate that circadian clock exerts important temporal control in distinct aspects governing stem cell behaviors [7, 8, 26, 27]. In skeletal muscle, the circadian clock is intimately involved in modulation of muscle stem cell myogenic properties in tissue growth processes, in addition to maintenance of nutrient metabolism and structural integrity [6, 28, 29]. A functional muscle clock is required to facilitate metabolic fuel switch from fat oxidation to glucose utilization as a nutrient sensor that determines insulin sensitivity by responding to feeding signals [30]. The metabolic role of the muscle clock was also highlighted by recent findings of augmented metabolic rhythm by exercise regimen [29, 31]. Our previous studies revealed that the clock is required to orchestrate myogenic progression of muscle stem cells [32–35]. The concerted clock facilitates myogenic progenitor differentiation into mature multi-nucleated myotubes, a process that involves both the positive and negative regulatory arms of the circadian clock transcriptional feed-back regulatory circuit. Bmal1 deficiency impairs myogenic differentiation and regenerative myogenesis, whereas ablation of its transcription repressor Rev-erbα promotes these processes [32, 33, 36]. In addition, Per1/Per2 and Cry2, key regulators within the negative molecular clock loop, modulate myogenesis that impacts muscle regeneration [34, 35]. Moreover, loss of *Bmal1* resulted in obesity with reduced muscle mass [32, 37]. Thus, targeting the circadian clock function, based on its modulation of myogenic progenitors in skeletal muscle, may be of potential therapeutic use to promote regenerative capacity in dystrophic or degenerative muscle diseases.

Current efforts in discovering clock modulators are focused on metabolic diseases and cancer therapy. Agonists for ligand-binding nuclear receptors Rev-erbα and ROR displayed metabolic benefits [24, 38–40], while Cry-stabilizing molecules may have utility in Glioblastoma Multiforme [13, 14, 25]. At present, small molecules directly targeting the key transcriptional driver of the core clock loop, the CLOCK protein, have yet to be uncovered. In the current study, we conducted a screen for compounds specifically targeting the CLOCK protein to modulate circadian clock activity and myogenesis. Applying this screening platform, we identified chlorhexidine (CHX) as a novel clock activator with pro-myogenic activities that may have potential muscle disease utilities.

## Materials and methods

### Cell culture

The sources of the cell lines used in the study are listed in Suppl. Table 1. Cells were maintained at 37 °C in 10% fetal bovine serum (FBS, Cytiva) with 1% Penicillin–Streptomycin-Glutamine in Dulbecco's Modified Eagle Medium (DMEM, Thermo Fisher Scientific). Cells were split via digestion using 0.25% Trypsin–EDTA (Thermo Fisher CN25200072). 80–90% confluent cultures of C2C12 myoblasts were subjected to myogenic differentiation using 2% FBS-supplemented DMEM.

### All-around molecular docking-based screening pipeline

Briefly, the protein crystal structure of CLOCK was obtained from RCSB Protein Data Bank (PDB 4f31 [20]. An in-house developed Ligand Virtual Screening Pipeline (LiVS), as described [41, 42], was employed to screen in silico the NCI Developmental Therapeutics Program (DTP) compound library (containing about 260,000 compounds) and the available FDA library to identify structural hits. LiVS method is a multiple-stage and full-coverage pipeline for ligand screening that utilizes the three precision modes (i.e., HTVS, high-throughput virtual screening; SP, standard precision; and XP, extra precision) of Schrodinger Glide software [43] for docking analysis. First, a HTVS precision mode, which is fast but less accurate, was implemented to dock the entire library. The 10,000 top-ranked compounds were next docked and scored by the SP mode. Then the 1000 top-ranked compounds from SP precision docking were re-docked and re-scored by the XP mode. The 1000 compounds were further analyzed and filtered by Lipinski’s rule of five [44], HTS frequent hitter (PAINS) [45], protein reactive chemicals such as oxidizer or alkylator (ALARM) [46]. Molecule diversity were maximized using Universal Diversity Score, a method developed to measure library diversity independent of library size. Based on the virtual screening pipeline, we requested the top 266 compounds from NCI-DTP and obtained 83 available for secondary functional screening.

### Luminometry, real-time bioluminescence recording and data analysis

U2OS cells containing a stable Period2 promoter-driven luciferase (*Per2::dLuc*) reporter [47, 48] were seeded at 4 × 10^5^/well onto 24 well plates and treated at 90% confluence with fresh explant medium with compounds at indicated concentration. The optimal seeding density used was based on 90% confluence at 24 h after seeding. Fresh explant medium contains 1M pH7 HEPES (Thermo Fisher 15630080), 7.5% sodium bicarbonate (Thermo Fisher 25080094), 100 mM sodium hydroxide (Fisher Chemical S318-500), 100 mM XenoLight D-Luciferin monopotassium salt bioluminescence substrate (PerkinElmer 122799) with 10% FBS and 1% PSG in DMEM. Plates were sealed with plastic film and placed inside a LumiCycle96 (ActiMetrics, Wilmette, IL, USA) for luminometry, kept in a bacterial incubator at 36^0^C without CO2. Cells were maintained for 7 days for luciferase luminescence activity recording. Measurement of bioluminescence rhythms from *Per2::dLuc* U2OS reporter cell line was conducted as described [47, 49, 50]. Briefly, luminescence from each well was measured for ~ 70 s at intervals of 10 min and recorded as counts/second. Raw and subtracted results of real-time bioluminescence recording data for 6 days were exported, and data was calculated as luminescence counts per second. The LumiCycle Analysis Program (ActiMetrics) was used to determine clock oscillation period length, amplitude and phase. Raw data following the first cycle from day 2 to day 5 were fitted to a linear baseline, and the baseline-subtracted data (polynomial number = 1) were fitted to a sine wave, from which period length and goodness of fit and damping constant were determined. For samples that showed persistent rhythms, goodness of fit of > 80% was usually achieved.

### Plasmids and shRNA

pcDNA3-BMAL1-His plasmid was purchased from Addgene (Addgene 31367). pcDNA3.0-6XMyc-Clock and pcDNA3.0-3XFLAG-Cry2 were generated by PCR amplification and sub-cloned into the pCDNA3.0–6XMyc or pCDNA3.0–3XFLAG vector separately. The primers used for PCR amplifications were: CLOCK-s: GCGGCCGCATGGTGTTTACCGTAAGC, CLOCK-as: CTCGAGTTCAGCCCTAACTTCTGCA; Cry2-s: GCGGCCGCATGGCGGCGGCTGCTGTGGT Cry2-as: CTCGAGTCAGGAGTCCTTGCTTGCT. The cysteine 267 mutation of *CLOCK* gene was generated using site-directed mutagenesis. The primer sequences used to generate this mutation were: *CLOCK*-C267A-s: TTCATCAAGGAAATGGCTACTGTTGAA, *CLOCK*-C267A-as: GCCATTTCCTTGATGAACTGAGGTG. The short hairpin RNAs of CLOCK used to generate stable knockdown U2OS cells were purchased from Sigma-Aldrich (TR0000018976) and cloned into the PLKO.1 puro vector.

### Generation of stable cell lines

Stable clones of U2OS cells containing control or sh*CLOCK* were generated by lentiviral transduction and stable selection. HEK293A were transfected with lentiviral packaging plasmids (psPAX2 and pMD2.G) and lentivirus vectors PLKO.1 or PLKO.1-sh*CLOCK* using PEI Max. At Forty-eight hours after transfection, lentiviruses were collected through a 0.45-µm filter to remove cell debris. U2OS cells were then infected using the lentiviral medium supplemented with polybrene (Santa Cruz, SC-134220). Stable cell colonies were selected in the presence of 1 μg/ml puromycin at 24 h following lentiviral infection,

### Transient transfection luciferase assay

Luciferase assays were performed as described [51]. Cells were seeded at 4 × 10^5^/well on 24 well plates. At 90% confluence, cell transfection was performed using PEI max reagent (Polysciences 24765-1) in Opti-MEM Reduced Serum Medium (Thermo Fisher 31985062) with plasmid constructs, including PGL2, PRL, PcDNA3.0-6XMyc-CLOCK (Addgene 47334), pcDNA3.0-BMAL1-His (Addgene 31367), and pcDNA3.0-3XFLAG-Cry2. Twenty-four hours after transfection, cells were treated with test compounds in 10% FBS 1% PSG DMEM media for indicated times. Luciferase assay was carried using the Dual-Luciferase Reporter Assay Kit (Promega E1910) according to manufacturer’s protocol. Luminescence was measured using a microplate reader (TECAN infinite M200pro). The mean and standard deviation values were calculated for each well and graphed.

### RNA extraction and quantitative reverse-transcriptase PCR analysis

RNA extraction was performed using Trizol reagent (Ambion 15596018). RNA concentration was measured using Nanodrop spectrophotometer (Thermo Fisher). Reverse transcription was carried out using a Revert-Aid RT Reverse Transcription Kit (Thermo Fisher K1691) and run on a SimpliAmp Thermal Cycler (Thermo Fisher A24811). Quantitative PCR was performed using SYBR Green Master Mix (Thermo Fisher A25742) using the ViiA 7 Real-Time PCR System (Applied Biosystems). Primer sequences used are specified in Supplemental Table 2. Relative expression levels were determined using the comparative CT method to normalize target genes to 36B4 internal controls.

### Immunoblot analysis

Total protein (20–30 µg) was extracted using immunoprecipitation lysis buffer (3% NaCl, 5% Tris–HCl, 10% Glycerol, 0.5% Triton X-10 in Milli Q water) and resolved on 10% SDS-PAGE gels followed by western blotting on Immuno-Blot PVDF membranes (Bio-Rad). Antibodies used were diluted in 5% milk. Membranes were washed with TBST containing 10% TBS and 0.1% Tween 20 (Fisher Chemicals BP377-500). Images were developed by Super Signal West Pico PLUS Stable Peroxide Solution (Thermo Scientific 1863095) and Enhancer Solution (Thermo Scientific CN: 1863094) using a chemiluminescence imager (GE Bio-Sciences AB Amersham Imager 680). Primary and secondary antibodies used are described in Supplemental Table 3.

### Immunoprecipitation

HEK293A cells were transfected with pcDNA3.0-BMAL1-His, PcDNA3.0-6XMyc-CLOCK or PcDNA3.0-6XMyc-CLOCKC267A using Polyethylenimine PEI MAX reagent (Polysciences 247,651). Twenty-four hours after transient transfection, cells were treated with the indicated compounds for 8 h and homogenized in lysis buffer (50 mM Tris pH7.5, 150 mM NaCl 0.5% Triton X-100, 5% glycerol and protease inhibitor). Immunoprecipitation was performed overnight using Anti-c-Myc Agarose beads (Thermo Fisher 20168). Beads were washed three times with lysis buffer. The resultant protein samples were resolved by SDS-PAGE and immunoblotted with antibodies that are described in Supplemental Table 3.

### Myogenic differentiation of C2C12 myoblasts

C2C12, C2C12 Bmal1 knockdown (BMKD) and scrambled control (SC) myoblasts were seeded (1–2 × 10^5^/well) onto 6-well tissue culture plates and maintained in 10% FBS until 90% confluency. DMEM media containing 2% FBS was used to induce differentiation as day 0, with treatment of indicated compounds or DMSO control. Daily phase-contrast images were obtained to monitor morphological changes. Proteins and RNA samples were collected on the indicated days.

### Myosin heavy chain immunofluorescence staining

C2C12, C2C12 Bmal1 knockdown and scrambled control myoblasts were seeded at 2 × 10^5^/well onto 12-well plates and maintained in 10% FBS until 80% confluency. DMEM with 2% FBS was used to induce differentiation together with treatment of the indicated compounds. Myotubes were fixed with 4% paraformaldehyde on the indicated days after differentiation, permeabilized with 0.5% Triton X-100 (Fisher Bioreagents 9002-93-1) and blocked with 1% BSA (Fisher Bioreagents 9048-46-8). Primary antibodies (Additiona file 1: Table S4) were diluted in PBS solution with 0.01% Triton X-100 (Fisher Bioreagents 9002-93-1) and kept overnight at 4 °C. Cells were then incubated with secondary antibodies (Additiona file 1: Table S4) at room temperature for 1 h. 4′,6′-diamidine-2′-phenylindole dihydrochloride (DAPI,1 ug/ml) was used to label nuclei. Images were acquired using an Echo Revolve fluorescence microscope at 10X magnification. To quantify the proliferation of myoblast, cells were treated with 1 μg/ml of EdU for 3 h before fixation. Quantification of MHC-positive myonuclei and fusion index as indicated by the percentage of fused myotubes with 2 or more myonuclei was calculated based on six representative fields at 10× magnification.

### EdU proliferation assay

Cells were seeded at 0.2 × 10^5^/well in 12-well plates and treated with the indicated compounds and controls overnight. Incorporation of 5-ethynyl-2′-deoxyuridine (EdU, 10 μM) was analyzed following addition for 4 h according to the manufacturer’s protocol. Detection of EdU was performed using Click-iT EdU Imaging Kit with Alexa Fluor 488 (Invitrogen). DAPI (1 μg/ml) was used for labeling of nuclei. Images were acquired using an Echo Revolve fluorescence microscope at 20X magnification. The total number of EdU+ cells was determined using 10 representative fields, and the rate of proliferation was calculated as percentage of EdU^+/^DAPI.

### Wound healing assay

Cells were seeded at 12 × 10^5^/well onto 6-well plates until 90% confluent and treated with compounds as indicated, while maintained in media containing 10% FBS 1% PSG DMEM. Scratch wounds were made with disposable cell scrapers and images were taken at 0, 4, 8, 24, and 48 h after scratch wound creation. Coverage of the wound surface at a given time point over the original wound area was used to determine the percent wound closure. Image J software (NIH, Bethesda, MD) was used for image quantification.

### Statistical analysis

Experiments results were analyzed and graphed using PRISM, and data were presented as mean + SD. Each experiment was repeated at least twice. Biological replicates were indicated for each experiment in the figure legends. Two-tailed Student's *t* test or one-way ANOVA with Tukey’s post hoc analysis for multiple comparisons was performed as appropriate. *P* < 0.05 was considered statistically significant.

## Results

### Screening pipeline to identify small molecule modulators of circadian clock

**Fig. 1:**
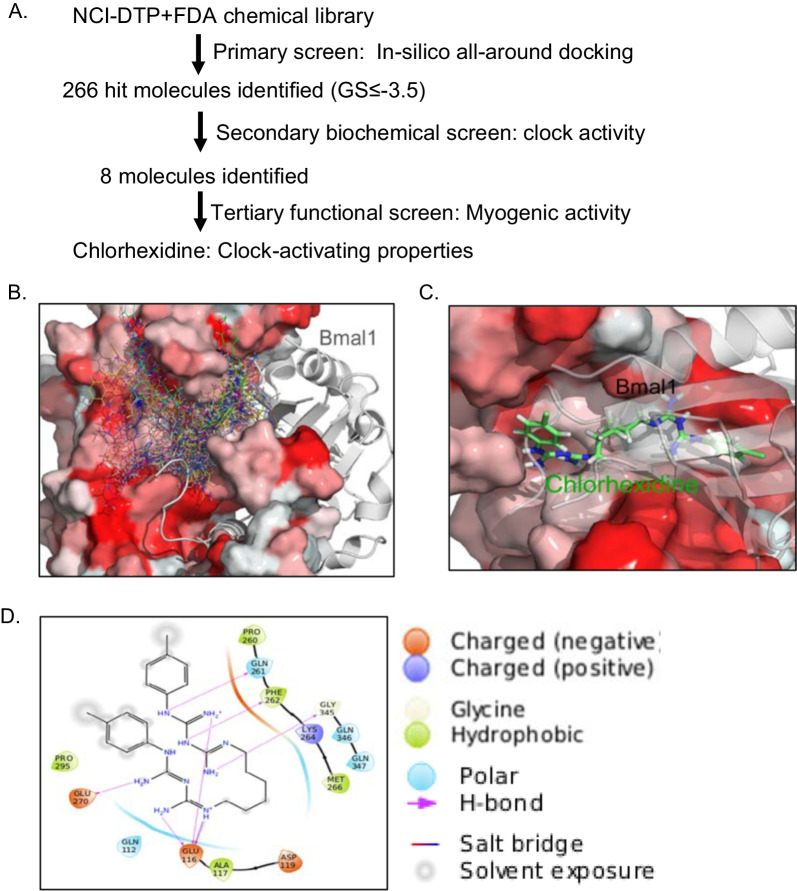
In silico screening and identification of chlorhexidine as a circadian clock modulator. **A** Screening and biochemical validation pipeline for identification of clock modulators using NCI/DTP-FDA chemical libraries. **B** Docking poses of 266 hit compounds identified via LiVS modeling targeting the CLOCK protein hydrophobic pocket that lies within the CLOCK-Bmal1 interaction interface. Crystal structure of heterodimeric CLOCK (red) with Bmal1 (gray) is based on PDB: 4f3l. **C** Docking pose of chlorhexidine (green) within the CLOCK protein hydrophobic pocket structure. CLOCK protein is shown in red and Bmal1 as gray. **D** Predicted interactions of chlorhexidine with key amino acid residues of the CLOCK hydrophobic pocket

Therapeutic targeting of circadian clock to date focused largely on repressor proteins in the molecular clock feed-back loop. To identify small molecules targeting the clock transcription activator CLOCK, we conducted an in silico molecular docking-based screening of chemical libraries from the NCI Developmental Therapeutic Program (DTP) with (~ 275,000 compounds) and the FDA library [4086 compounds]. The crystal structure of the CLOCK protein, PDB 4f31 [20], was used for molecular docking modeling for high-throughput virtual screening strategy to identify compounds with structural fit [42]. The screening strategy pipeline is shown in Fig. 1A. All-around docking (ADD) analysis identified hit molecules with ranked structural fit for a deep hydrophobic pocket we discovered, within the well-defined PAS-A domain of the CLOCK protein that mediates heterodimerization with its obligatory partner Bmal1 [20]. Compounds were ranked based on Glide Score calculation that predicts potential strength of binding with predicted hydrophobic, polar, and hydrogen bond interactions with key residues within this structure. Two hundred sixty-six compounds were identified as hits based on Glide Score ranking of ≤ 3.5. The docking poses are shown in Fig. 1B. Out of these initial hits from virtual screening, we were able to obtain 84 molecules for secondary screening via biochemical assays to determine their clock-modulatory activity. Employing a gold-standard assay to assess circadian clock properties with a U2OS luciferase reporter cell line containing a Period 2 gene promoter-driven luciferase construct (*Per2::dLuc*) [47], this biochemical clock function screen identified 8 molecules with significant modulation of clock activity. Chlorhexidine (CHX) was the only molecule that exhibited clock-activating properties. CHX displayed a strong GS score of ~ 19.5, with a tight docking pose within the targeted CLOCK hydrophobic pocket (Fig. 1C and Additiona file 1: Fig. S1A). Predicted interactions of CHX with the key amino acid residues lining the CLOCK pocket structure within 10-20A^0^, including charged, hydrophobic, polar, and hydrogen bonds, were indicated (Fig. 1D). A detailed analysis of theses interactions is shown in Additiona file 1: Fig. S1B.

### Identification of chlorhexidine as a circadian clock activator

**Fig. 2:**
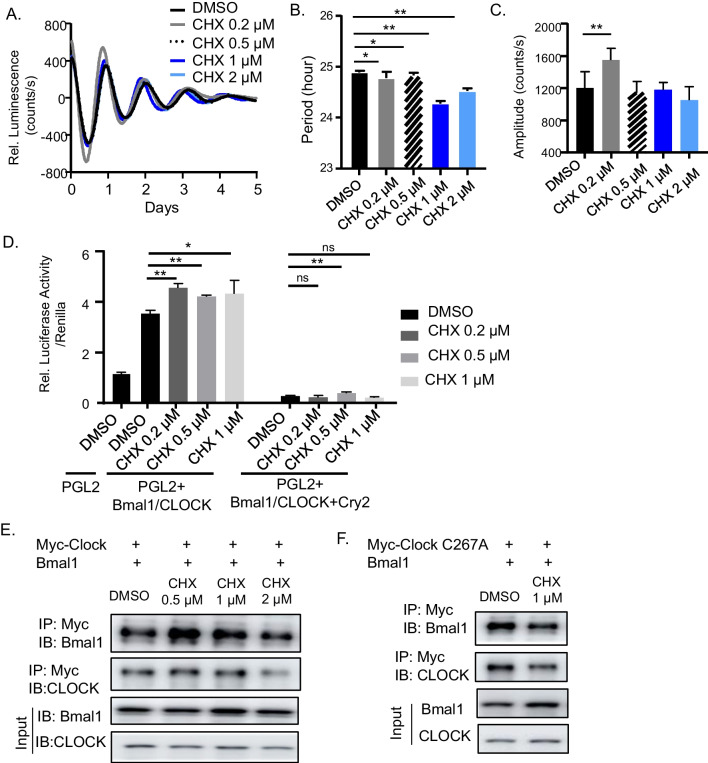
Biochemical characterization of chlorhexidine as a circadian clock activator. **A** Average tracing of bioluminescence activity monitoring of *Per2::dLuc* U2OS reporter cell line for 6 days using Lumicycle, with chlorhexidine treatment (CHX) at indicated concentrations as shown in baseline-adjusted plots. **B**, **C** Quantitative analysis of clock period length (**B**) and cycling amplitude (**C**) at CHX concentrations as indicated. Data are presented as Mean ± SD of *n* = 4 replicates for each concentration tested, with four independent repeat experiments. *, **p < 0.05 and 0.01 CHX versus DMSO by Student's *t* test. **D** Transient transfection luciferase assay using a Per2-driven luciferase reporter in U2OS cells with indicated CHX concentration. PGL2: empty vector control. *N* = 5 for each concentration tested. *, **p < 0.05 and 0.01 CHX versus DMSO by Student's *t* test. **E**, **F** Co-immunoprecipitation analysis of Bmal1 interaction with Myc-tagged CLOCK protein (**E**), or Myc-tagged C267A mutant CLOCK protein (**F**), with or without CHX treatment for 16 h at indicated concentrations in 293 T cells. Uncropped gels are shown in Additiona file 1: Fig. S5

The activities of CHX on modulating key properties of circadian clock function, including oscillating period length and amplitude, were determined via continuous monitoring of bioluminescence activity of the *Per2::dLuc* U2OS reporter cell line [47, 48]. Cells were treated with CHX at indicated concentrations (0.2 to 2 μM) at the start of the bioluminescence recording, as shown in original and baseline-subtracted plots for 6 days (Fig. 2A and Additiona file 1: Fig. S2A). Quantitative analysis revealed reductions of period length in CHX-treated versus DMSO control with the strongest effect observed at 1 μM (Fig. 2B). The effect of CHX on period length shortening revealed its ability to activate clock resulting in shorter cycles. In addition, CHX at 0.2 μM, but not at higher concentrations, induced increased clock cycling amplitude (Fig. 2C). Higher concentrations of CHX (5 and 10 μM) actually reduced amplitude suggesting toxicity (data not shown). To determine CHX had a direct effect on clock activation, we assessed the impact of acute CHX treatment on CLOCK/Bmal1-mediated transcription which we monitored using transient transfection to activate a Per2-luciferase reporter [48, 52]. As expected, Bmal1 and CLOCK co-transfection induced Per2-luciferase activity by ~ fourfold (Fig. 2D). CHX treatment at 0.2 μM for 6 h stimulated ~ 26% higher CLOCK/Bmal1-mediated transcription activation with similar effects observed at higher concentrations. Addition of Cry2 suppressed CLOCK/Bmal1-mediated transcription, as expected, while CHX showed moderate effect on augmenting luciferase activity under Cry2 repression at 0.2 μM.

Since our screening strategy focused on the CLOCK hydrophobic pocket involved in heterodimerization with Bmal1, identified compounds could impact this interaction to modulate CLOCK/Bmal1 transcriptional activity. We tested whether CHX activation of clock was mediated through promoting CLOCK/Bmal1 heterodimer formation to promote transcription. Using co-immunoprecipitation to detect CLOCK binding with Bmal1, we found that CHX (0.5 to 2 μM) augmented Bmal1 level immunoprecipitated by Myc-CLOCK (Fig. 2E). To test whether the effect of CHX on CLOCK/Bmal1 interaction depends on the CLOCK pocket structure, we mutated the key residue, cysteine 276 (C276), to alanine. This CLOCK C276 mutation did not affect its association with Bmal1 as compared to wild-type CLOCK with efficient Bmal1 detection in immunoprecipitated protein complex. However, CHX failed to increase the interaction between CLOCK C267 mutant and Bmal1, suggesting that CHX effect is dependent on the targeted CLOCK structure **(**Fig. 2F). Collectively, these results reveal that CHX is a novel circadian clock activator and functions by promoting CLOCK/Bmal1 interaction to activate transcription.

### Chlorhexidine induces clock gene expression

**Fig. 3:**
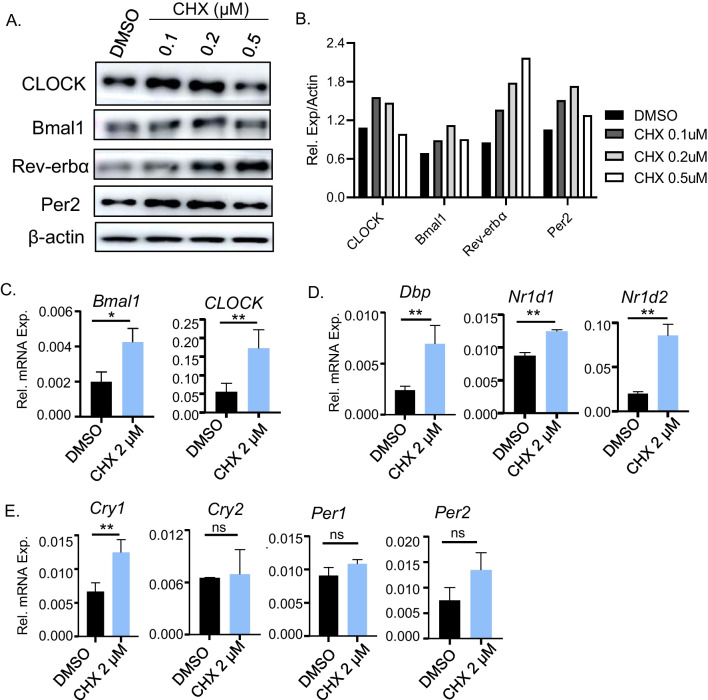
Effect of chlorhexidine on core clock gene expression in osteosarcoma cells. **A**, **B** Immunoblot analysis of core clock protein expression with CHX treatment at indicated concentrations for 24 h in U2OS cells (**A**) with quantitative analysis (**B**). Each lane represents pooled samples of 3 replicates. Uncropped gels are shown in Additiona file 1: Fig. S6. **C**–**E** RT-qPCR analysis of clock genes, including core clock activators (**C**), Bmal1/CLOCK target genes (**D**) and clock repressor genes (**E**), after CHX treatment for 24 h in U2OS cells. Data are presented as Mean ± SD of *n* = 3 replicates with two independent repeat experiments. *, **p < 0.05 and 0.01, CHX versus DMSO by Student's *t* test

Based on findings of CHX in stimulating CLOCK/Bmal1-mediated transcription, we determined its effect on inducing clock gene expression in U2OS cells. CHX at 0.1 and 0.2 μM induced CLOCK protein levels, with elevated Bmal1 expression at 0.2 and 0.5 μM (Fig. 3A, B). Rev-erbα and Period 2 (Per 2), direct transcription targets of CLOCK/Bmal1 in the core clock circuit, were also induced by CHX in a largely dose-dependent manner. CHX at 0.5 μM had an attenuated effect on these proteins except Rev-erbα. Consistent with this, CHX markedly upregulated *CLOCK* and *Bmal1* mRNA (Fig. 3C). CHX also increased *Dbp*, *Nr1d1*, *Nr1d2* and *Cry2* (Fig. 3D, E). As these components of the molecular clock are all transcriptionally controlled by CLOCK/Bmal1, these results further corroborated that CHX increases activity of CLOCK.

**Fig. 4:**
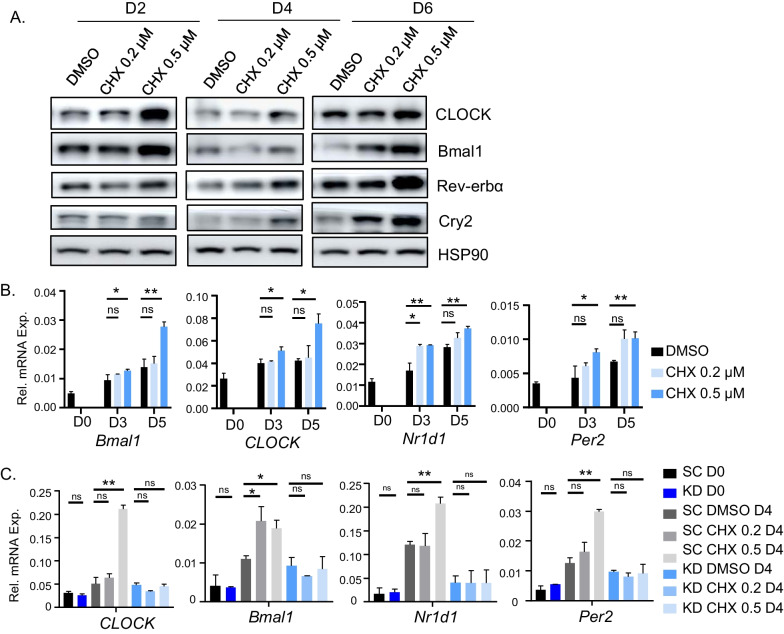
Effect of chlorhexidine on clock gene regulation in C2C12 myoblasts. **A** Immunoblot analysis of CHX effect on clock protein expression in C2C12 myoblasts during day 2, 4, and 6 of myogenic differentiation at indicated concentration. Each lane represents pooled samples of 3 replicates. Uncropped gels are shown in Additiona file 1: Fig. S7. **B** RT-qPCR analysis of CHX treatment on expression of clock genes at day 0, 3, and 5 of myogenic differentiation. **C** RT-qPCR analysis of expression of clock genes in C2C12 myoblasts with stable expression of scrambled control shRNA (SC) or Bmal1 shRNA (BMKD) in response to CHX treatment before and at day 4 of C2C12 myogenic differentiation. Data are presented as Mean ± SD of *n* = 3 replicates with three independent repeat experiments. *, **p < 0.05, and 0.01 CHX versus DMSO by Student's *t* test

Circadian clock components coordinate the progression of myogenic differentiation [6, 32, 33]. Using C2C12 mouse myoblasts, we determined whether CHX modulates clock activity in myogenic progenitors. During myoblast differentiation days 2, 4 and 6, CHX at 0.5 µM robustly stimulated CLOCK and Bmal1 protein levels, together with their transcriptional targets Rev-erbα and Cry2 (Fig. 4A and Additiona file 1: Fig. S2B). At day 6, Bmal1, Rev-erbα, and Cry2 protein were elevated in cells treated with 0.2 µM CHX. The augmented clock protein expression by CHX was corroborated by induction of these transcripts in a largely dose-dependent manner (Fig. 4B). Furthermore, using myoblasts containing stable shRNA silencing of Bmal1 (BMKD) as compared to cells with scrambled control (SC) [32], we tested whether CHX induction of clock gene expression is dependent on a functional clock. As expected, CHX induced *Clock*, *Bmal1* and their transcription targets, *Nr1d1* and *Per2* in SC myoblasts (Fig. 4C) at day 4 of myogenic differentiation. This effect was abolished in BMKD cells with loss of Bmal1 function. Thus, the effect of CHX on activating clock gene transcription is dependent on a functional clock.

### The clock-dependent effects of chlorhexidine on promoting myogenesis

**Fig. 5:**
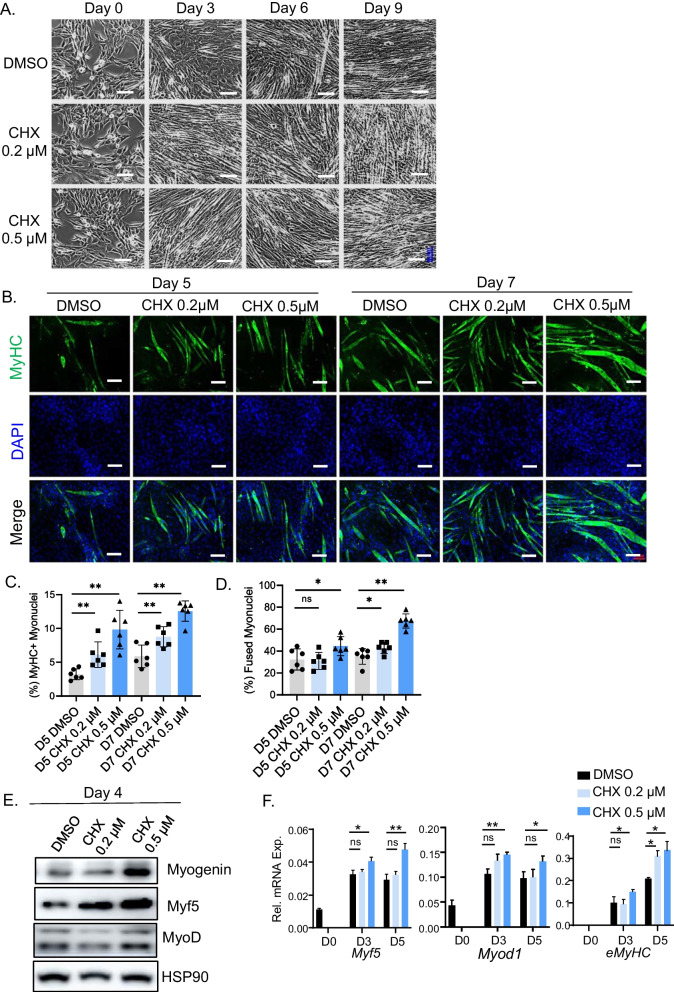
Effect of chlorhexidine on promoting C2C12 myogenic differentiation. **A** Representative phase-contrast images of C2C12 morphology on day 0, 3, 6, and 9 of myogenic differentiation with CHX treatment. **B**–**D** Representative images of myosin heavy chain (MyHC) immunofluorescence staining at day 5 and day 7 of myogenic differentiation (**B**), with quantification of percentage of MyHC^+^ myonuclei (**C**) and the fusion index as indicated by the percentage of myotubes with 2 or more fused myonuclei (**D**). Scale bar: 100 µm. **E**, **F** Immunoblot analysis (**E**), and RT-qPCR analysis of myogenic gene expression (**F**) at indicated CHX concentrations during C2C12 myogenic differentiation. Uncropped gels are shown in Additiona file 1: Fig. S8. Data are presented as Mean ± SD of *n* = 3 replicates with three independent repeat experiments. *, **p < 0.05 or 0.01 CHX versus DMSO by Student's *t* test

Our previous studies demonstrated that the essential clock activator, Bmal1, promotes myogenesis via direct transcriptional control of components of Wnt signaling [15, 32, 36]. We postulated that CHX, as a clock-activating molecule, may display pro-myogenic activity in myoblast differentiation. Phase-contrast images showed that, upon 2% serum induction for 9 days, normal C2C12 myoblasts differentiated efficiently into mature multi-nucleated myotubes, as expected (Fig. 5A). In comparison, CHX treatment at 0.2 or 0.5 μM markedly accelerated the morphological progression of myotube formation. Abundant mature myotubes formed at day 3 of early in response to CHX, whereas mature myotubes were barely visible in DMSO-treated cells. Myotube formation in day 3 CHX-treated cells was nearly complete, and comparable to the differentiation observed at day 6 of control cells, indicating that CHX enhanced myocyte maturation. This effect was maintained throughout the differentiation time course, with CHX-treated cells displaying more advanced myotube elongation at day 6 and 9 as compared to controls. However, higher concentrations of CHX (2 and 5 μM) appeared to be toxic to myoblasts with significant cell death during differentiation. Next, we assessed the pro-myogenic activity of CHX using immunofluorescence staining for a mature myocyte marker, myosin heavy chain (MyHC). Consistent with the advanced morphological progression, CHX treatment increased numbers of MyHC-positive mature myotubes at day 5 and day 7 of differentiation (Fig. 5B, C), and 0.5 µM CHX induced notable myotube hypertrophy at day 7. Quantification of fusion index revealed significantly elevated myogenic fusion by CHX (Fig. 5D), a hallmark of mature myogenic progression. CHX effect on promoting myogenic maturation was lost in myoblasts without a functional clock. This effect was evident as early as at day 3 of differentiation (Additiona file 1: Fig. S3). In line with the augmented myogenic differentiation, CHX led to early induction of myogenic factors, Myf5 and Myogenin proteins, at day 3 and day 5 (Fig. 5E). *Myf5*, *Myod1,* and *embryonic myosin heavy chain (eMyhc)* transcripts were also significantly upregulated by 0.5 µM CHX with a tendency toward higher expression at 0.2 µM (Fig. 5F). The differentiated myocyte markers, Myogenin and myosin light chain, were similarly induced by CHX (Additiona file 1: Fig. S3C).

**Fig. 6:**
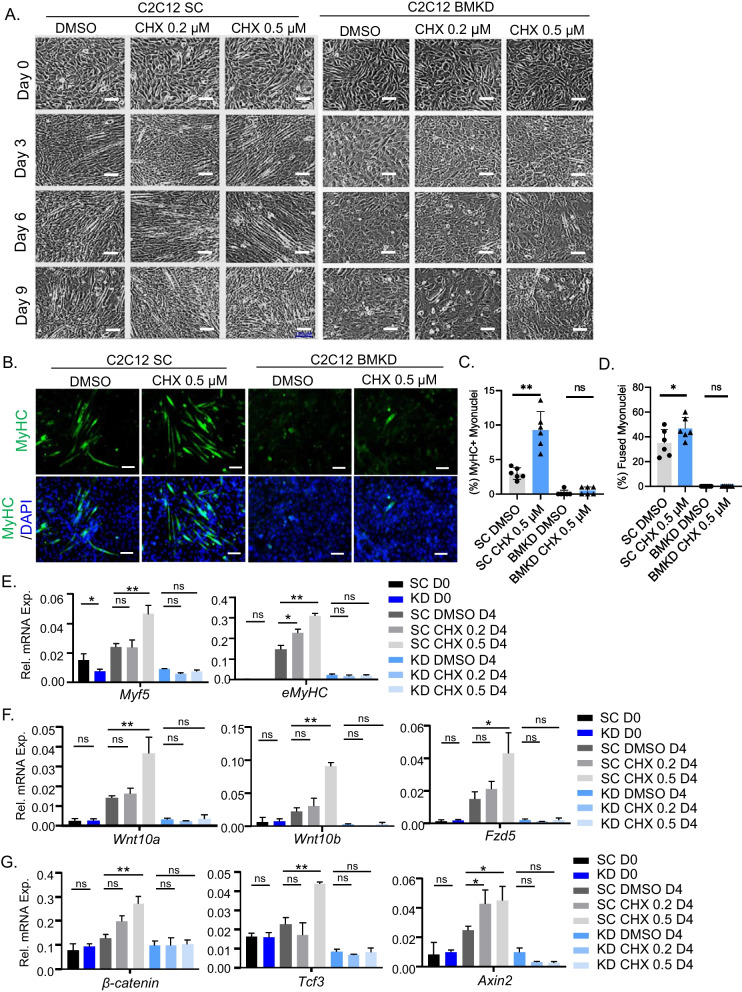
Clock-dependent effect of chlorhexidine on myogenesis. **A** Representative phase-contrast images of myogenic differentiation morphology at day 0, 3, 6, and 9 in response to CHX treatment in C2C12 myoblasts with stable scrambled control shRNA (SC) or Bmal1 shRNA (BMKD). Scale bar: 100 μm. **B**–**D** Representative images of myosin heavy chain (MyHC) immunofluorescence staining at day 5 of myogenic differentiation in SC and BMKD myoblasts, with quantification of percentage of MyHC + myonuclei (**C**) and the fusion index as indicated by the percentage of myotubes with 2 or more fused myonuclei (**D**). Scale bar: 100 µm. **E**–**G** RT-qPCR analysis of myogenic gene expression (**E**), and genes of Wnt pathway (**F**, **G**) in SC and Bmal1 BMKD C2C12 myoblasts in response to CHX treatment before and at day 4 of C2C12 myogenic differentiation. Data are presented as Mean ± SD of *n* = 3 replicates with two independent repeat experiments. *, **p < 0.05 or 0.01 CHX versus DMSO by Student's *t* test

Next, we examined the pro-myogenic activity of CHX using BMKD myoblasts to determine whether this effect is dependent on clock modulation. Due to inhibition of Bmal1, myogenic differentiation of BMKD myoblasts was impaired as compared to SC control cells (Fig. 6A), as previously reported [32]. Similarly, CHX stimulated myotube formation of SC myoblasts as observed for the parental C2C12 myoblasts, albeit the rate of myogenic progression was attenuated (Fig. 6A). In contrast, CHX failed to augment BMKD myoblast differentiation, indicating that its pro-myogenic activity is clock dependent. MyHC staining in these cells at day 5 of differentiation revealed the loss of CHX effect on enhancing myocyte maturation in the BMKD myoblasts as compared to the strong effect observed in SC cells (Fig. 6B). Quantitative analysis of MyHC^+^ myonuclei (Fig. 6C) and fusion index (Fig. 6D) corroborated that CHX effect on promoting myogenic maturation was lost in myoblasts without a functional clock. Gene expression analysis confirmed the clock-dependent effect of CHX on inducing the myogenic program, with loss of CHX induction of *Myf5* and *eMyhc* in Bmal1-deficient myoblasts (Fig. 6E). During myogenesis, clock exerts direct transcriptional control on genes involved in disparate steps of Wnt signaling pathway [32]. We examined the known transcriptional targets of clock in this pathway and found that CHX stimulated upstream Wnt ligands including Wnt10a and Wnt10b, and the Wnt receptor Frizzled 5 (Fzd5). In addition, CHX induced the expression of β-catenin, a key effector of Wnt signaling, transcription factor TCF3, and Axin 2, the Wnt target gene, in SC myoblasts (Fig. 6F, G). These effects were abolished in Bmal1-deficient cells, indicating clock-dependent actions of CHX in activating a key signaling mechanism that promotes myogenic differentiation.

### Chlorhexidine promotes myoblast proliferation and migration

**Fig. 7:**
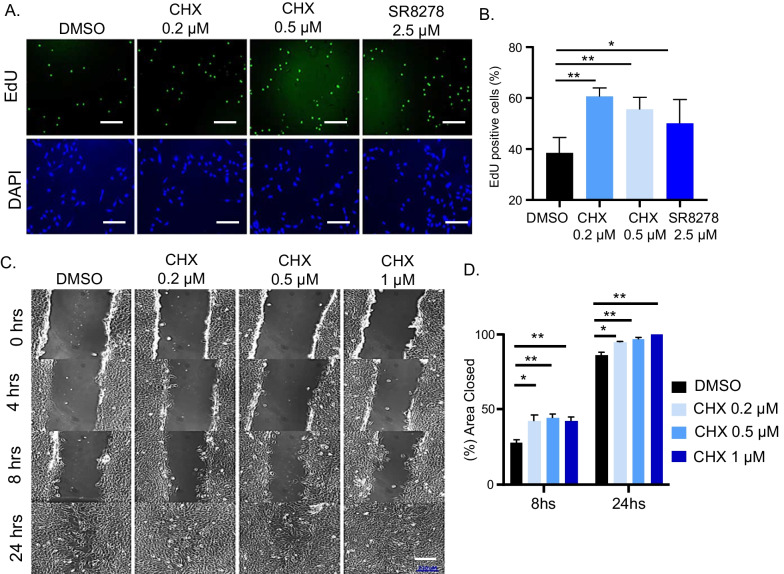
Effect of chlorhexidine on promoting myoblast proliferation and migration. **A**, **B** Representative images of EdU staining of C2C12 myoblasts with CHX or SR2878 treatment for 24 h (**A**), with quantitative analysis (**B**). 10 representative fields were used for quantitative analysis. *, **p < 0.05 and 0.01 treated versus DMSO by Student's *t* test. Scale bar: 100 µm. **C**, **D** Representative images of wound healing assay of C2C12 myoblasts for 24 h with CHX treatment at indicated concentrations (**C**) with quantitative analysis (**D**). 10 representative fields for each concentration. *, **p < 0.05 and 0.01, CHX versus DMSO by Student's *t* test. Scale bar: 230 µm

Muscle myogenic progenitors provide the major cellular source for tissue regeneration. Their activation, proliferative expansion and migratory activity in response to injury are key processes involved in muscle repair [53–55]. We determined potential effects of CHX on the proliferative and migrative activities of myoblasts. CHX at both concentrations tested induced ~ 40% higher proliferation as compared to DMSO (Fig. 7A, B). Furthermore, based on our previous findings of a Rev-erbα antagonist SR8278 on promoting myoblast proliferation [33], we included this clock modulator as a control. In comparison, CHX at 0.2 µM was more efficacious to stimulate myoblast proliferation than SR8278 at 2.5 µM (Fig. 7B). CHX also augmented proliferation in U2OS cells (Additiona file 1: Fig. S4A and S4B). The dose-dependent effect of CHX on promoting U2OS proliferation was dependent on a functional clock, as CHX-mediated proliferation was abolished in cells with *CLOCK* gene silencing (Additiona file 1: Fig. S4C and S4D). In a wound healing assay, treatment of C2C12 myoblasts with CHX (0.2 to 1 µM) significantly promoted wound closure in at 8 and 24 h as compared with DMSO, indicative of enhanced migration (Fig. 7C, D). Together these analyses indicate that in addition to its effect on promoting myogenic differentiation, CHX also augmented the proliferative and migratory activities of myoblasts that may contribute to regenerative repair.

## Discussion

Circadian clock disruption leads to the development of obesity, insulin resistance and various types of cancer [56, 57]. Re-enforcing clock regulation may yield metabolic benefits and has potential for anti-cancer therapies. Despite the current interests in developing clock modulators, compounds that directly modulate CLOCK activity remains to be uncovered. The screen platform described herein targeting the CLOCK protein led to the identification of CHX as a novel circadian clock-activating molecule that augments myoblast differentiation, proliferation and migration. Given that coordinated circadian clock control is required for muscle stem cell regulation in regenerative repair and protection against dystrophic muscle damage, CHX may have therapeutic utilities in dystrophic diseases and muscle-wasting conditions.

We leveraged a powerful high-throughput in silico screening platform based on molecular docking analysis of ~ 300 K compounds from NCI-DTP and FDA libraries to identify molecules with a structural fit for a hydrophobic pocket within the PAS-A domain of the CLOCK protein. Secondary functional screening to determine circadian clock properties of the top hits from initial in silico screen led to the selection of compounds with clock-modulatory activities. Lastly, biochemical validation of selected compounds for their modulation of CLOCK/Bmal1-mediated gene transcription and heterodimer interaction were performed. CLOCK forms an obligatory dimer with Bmal1 to activate transcription, and the PAS-A/B domains are structural elements mediating CLOCK and Bmal1 interaction. The docking of CHX with the CLOCK crystal structure revealed interactions with several key residues that form a deep hydrophobic pocket within the PAS-A domain. As CHX displayed clock-activating effect in a Per2-Luc reporter cell line, we postulated that its binding within the CLOCK hydrophobic pocket may enhance interaction affinity with Bmal1 to promote CLOCK/Bmal1-mediated transcription. Co-immunoprecipitation revealed increased CLOCK and Bmal1 association in cells treated with CHX, with induction of clock genes consistent with its clock-activating activity. As a lead candidate, the structure of CHX could be used to inform development of derivatives with improved efficacy toward clock modulation.

Recent endeavors to identify clock-modulating compounds focused on Rev-erbα, RORα and Cry. Rev-erbα/β and RORs are ligand-binding nuclear receptors and are readily “druggable” by targeting their ligand-binding pockets [23, 24, 38, 58, 59]. So far, the identified Cry modulators stabilize the protein and augment its clock repressor function by inhibiting its proteosome-mediated degradation [14, 25, 60]. To date, clock-modulating compounds that directly target CLOCK and Bmal1, the key transcription activators that drive core clock transcription feed-back loop have yet to be identified. This study is the first to report a CLOCK protein-targeting molecule, CHX, with activity in promoting myogenic differentiation. We provided experimental evidence for the target specificity of CHX, with loss of its clock-modulatory activity in clock mutant and its pro-myogenic effect in clock-deficient myoblasts. Mutating CLOCK protein cysteine 267 abrogated CHX-induced CLOCK/Bmal1 association, demonstrating the targeting specificity of the compound. Future experiments are needed to determine the binding affinity and specificity of CHX with CLOCK. Nonetheless, by testing CHX activity using CLOCK mutant protein, Bmal1-deficient myoblasts and CLOCK knockdown U2OS cells, current findings indicate that CHX activity in stimulating clock gene induction, myogenic differentiation and proliferation requires a functional clock.

CHX is a cationic bisbiguanide molecule capable of binding to the bacterial cell wall with broad bacteriostatic or bactericidal activity [61]. It is used as a topical skin antiseptic agent and in mouth wash due to its anti-plaque and anti-gingivitis activity [62, 63]. CHX binds to proteins on skin and mucous membranes with limited systemic absorption. Potentially due to its chemical properties, we found that CHX, at concentrations higher than 1–2 µM, exhibited toxicity, although distinct cell types showed variable sensitivity to CHX concentration and duration of treatment. Dose response analysis of bioluminescence activity using Per2-Luc U2OS cells revealed a broader nontoxic concentration range (0.2–2 μM) than functional assays of pro-myogenic activity in C2C12 myoblasts (0.2–0.5 µM). We confined experiments within the lower concentration range to avoid adverse effects. This contrasts with other studies for clock modulator screening that typically used 0.5–10 μM [50] or investigation of biological functions of clock-targeting compounds [24]. Nonetheless, CHX could have clock-independent off-target effects of CHX, particularly as related to pro-myogenic activities in myoblasts. Despite its significant pro-myogenic action, the observed clock-modulatory effects of CHX on period lengthening were relatively moderate. This could be due to its mechanism of action by promoting CLOCK interaction with Bmal1. As our results revealed that CHX augmented the affinity of these interacting partners, there could be a limit posed by the binding kinetics of these proteins in vivo, and thus, not as efficacious as inhibitors that disrupt endogenous interaction. In line with this, the clock-inhibitory molecules we discovered from this screen were more potent in inhibiting Bmal1/CLOCK interaction and altering period length (unpublished data). These observations highlight the need for optimization of CHX chemical structure in order to develop new efficacious clock-activating molecules with pro-myogenic activities, while minimizing toxicity and off-target effects. Optimization of this lead compound may lead to development of novel agents for dystrophic muscle disease or metabolic disease applications.

Current pharmacological modulators of circadian clock components are directed toward metabolic diseases and cancer [14, 19]. Epidemiological investigations and experimental studies have provided compelling evidence that disruption of clock regulation leads to the development of obesity and insulin resistance [64]. We reported that loss of Bmal1 resulted in obesity [37], and chronic shiftwork induced adipose tissue expansion with inflammation [65]. The activity of CHX in promoting clock function, conceivably, could be applied to metabolic disease treatment. A number of small molecule clock modulators demonstrated efficacy in metabolic disease models. Rev-erbα agonists SR9009 and SR9011, displayed anti-obesity and anti-hyperlipidemia actions [24, 58]. Nobiletin, a ROR agonist, promoted mitochondrial oxidative capacity in skeletal muscle that ameliorated insulin resistance upon a high fat diet challenge [39, 40]. It is conceivable that small molecules directly targeting CLOCK, such as CHX or similar agents, may have therapeutic efficacy toward mitigating obesity and Type II diabetes. The metabolic activities of CHX awaits further study.

The pro-myogenic properties of CHX were revealed based on our prior studies of circadian clock regulation in myogenesis. Both positive and negative arms of the clock transcriptional loop modulate myogenic differentiation, with Bmal1 promoting whereas Rev-erbα inhibiting this process [6, 15, 16, 32, 33, 66]. Notably, these regulations impact chronic dystrophic muscle damage in the dystrophin-deficient mdx mice, a preclinical model for Duchene Muscular Dystrophy [15, 16]. Bmal1 promotion of regenerative myogenesis is required to prevent dystrophic muscle damage [15], whereas loss of Rev-erbα ameliorates dystrophic pathophysiology [16]. Moreover, pharmacological inhibition of Rev-erbα by SR8278 attenuated the dystrophic pathophysiological changes in the mdx mice [67]. These data provided proof-of-principle that targeting clock components may ameliorate dystrophic disease. In line with this notion, CHX activation of clock in myoblasts resulted in enhanced differentiation and this effect was clock dependent. Moreover, CHX augmented the proliferation and migration of myoblasts, implicating its potential utility to facilitate disparate stages of muscle regenerative repair to ameliorate dystrophic disease. This study together with our prior findings suggests that clock modulators have potential utilities in muscle diseases, such as muscular dystrophy or muscle-wasting condition associated with chronic inflammation or aging. On the other hand, by activating clock, CHX also stimulated proliferation of myoblast. This finding implicates a potential undesirable effect of CHX on cellular proliferation that may increase the risk for tumorigenesis, and we indeed observed a similar effect on inducing U2OS cell proliferation. Developing new compounds that dampen the proliferative activity to reduce this adverse effect is a pre-requisite for future drug development. Future studies in preclinical models of muscle diseases to test both the in vivo efficacy and adverse effects of CHX or its derivatives are warranted.

## Conclusions

In summary, our study is the first report to demonstrate the feasibility of a screening pipeline for discovering circadian clock modulators by targeting the CLOCK protein that has myogenic regulatory properties. The discovery of CHX as a pro-myogenic molecule targeting the clock modulation may have therapeutic applications in muscular dystrophy or related muscle remodeling processes involving muscle stem cells.

## Supplementary Information

**Additional file 1**. Supplemental Figures S1–S4, Raw Data Figures S5–S8, and Supplemental Tables 1–4.

## Data Availability

All data generated and analyzed during this study are included in this published article and associated Supplementary Information files.

## Abbreviations

CLOCK: Circadian Locomotor Output Cycles Kaput
Bmal1: Brain and Muscle Arnt-like Protein 1
PAS: Per-Arnt-Sim
ROR: RAR-related Orphan Receptor
CHX: Chlorhexidine
MyHC: Myosin heavy chain
