# Supplementary material for: Targeted screening and identification of chlorhexidine as a pro-myogenic circadian clock activator
Source: Stem Cell Res Ther. 2023 Jul 31;14:190. doi: 10.1186/s13287-023-03424-2 (PMC10391781; doi:10.1186/s13287-023-03424-2)
Supplement: Supplementary file 1 — Additional file 1. Supplemental Figures S1–S4, Raw Data Figures S5–S8, and Supplemental Tables 1–4. [file 13287_2023_3424_MOESM1_ESM.pdf]

Supplemental Figure S1.

A.

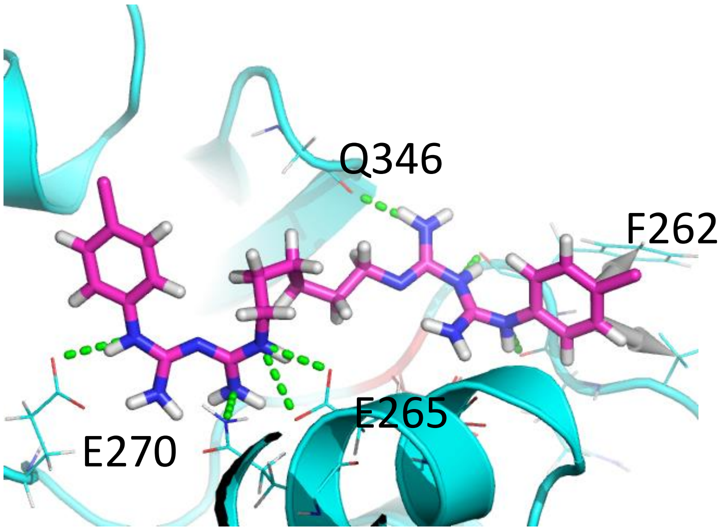

B.

Predicted Distance/Interaction	CLOCK AA Residue Position
≤3A	Q112 E116 A117 D119 Q261 F262 K264 E270 P295 G345 Q346 Q347
≤4A	P260 M266
≤6A	L113 L115 L118 L141 R256 I263 T268 E271 E274 P294 I296 G298 T343 K344
Hydrogen Bond	E116 Q261 F262 E270 G345 Q112
Hydrophobic interaction	PHE262, PRO260

12 Residues within 3A: Q112 E116 A117 D119 Q261 F262 K264 E270 P295 G345 Q346 Q347
2 Residues within 4A : P260 M266
14 Residues within 6A: L113 L115 L118 L141 R256 I263 T268 E271 E274 P294 I296 G298 T343 K344
6 unique residue for Hydrogen Bonds: E116 Q261 F262 E270 G345 Q112
2 Hydrophobic interaction pairs at 5A: PHE262, PRO260

Figure S1. Chlorhexidine interactions with residues within the CLOCK protein hydrophobic pocket. (A) Docking pose of CHX, represented as the pick stick molecule, on CLOCK hydrophobic pocket structure with interacting amino acid residues listed. (B) List of predicted CHX interactions with amino acid residues within the CLOCK hydrophobic pocket by all-around docking modeling.

Supplemental Figure S2.

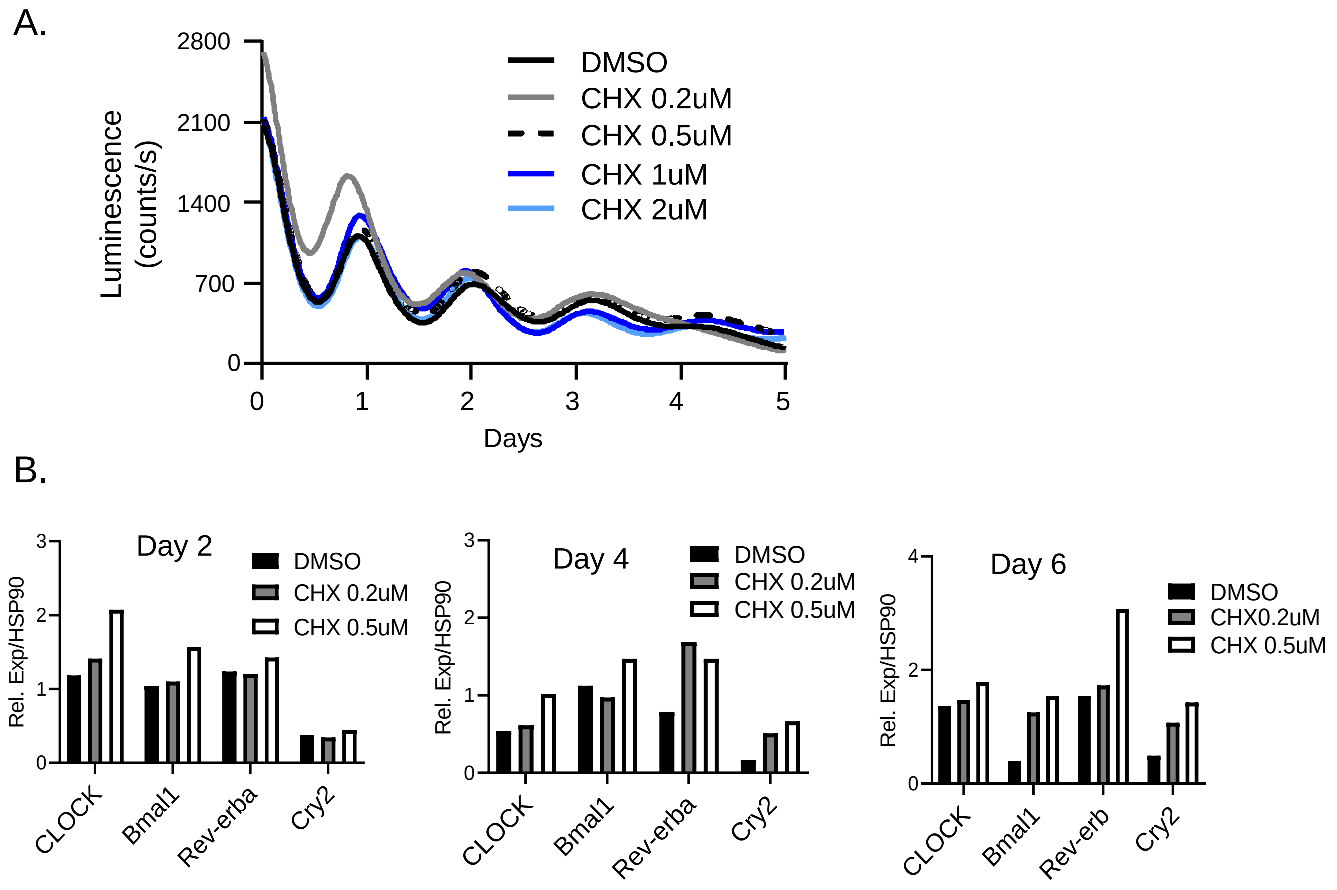

Figure. S2. (A) Original plot of average tracing of bioluminescence activity monitoring of Per2::dLuc U2OS reporter cell line for 6 days using Lumicycle, with Chlorhexidine treatment (CHX) at indicated concentrations as shown in baseline-adjusted plots. N=4/treatment. (B) Quantification of immunoblot analysis shown in Fig. 4A of Chlorhexidine effect on clock protein expression levels at day 2, 4 and 6 of myogenic differentiation.

Supplemental Figure S3.

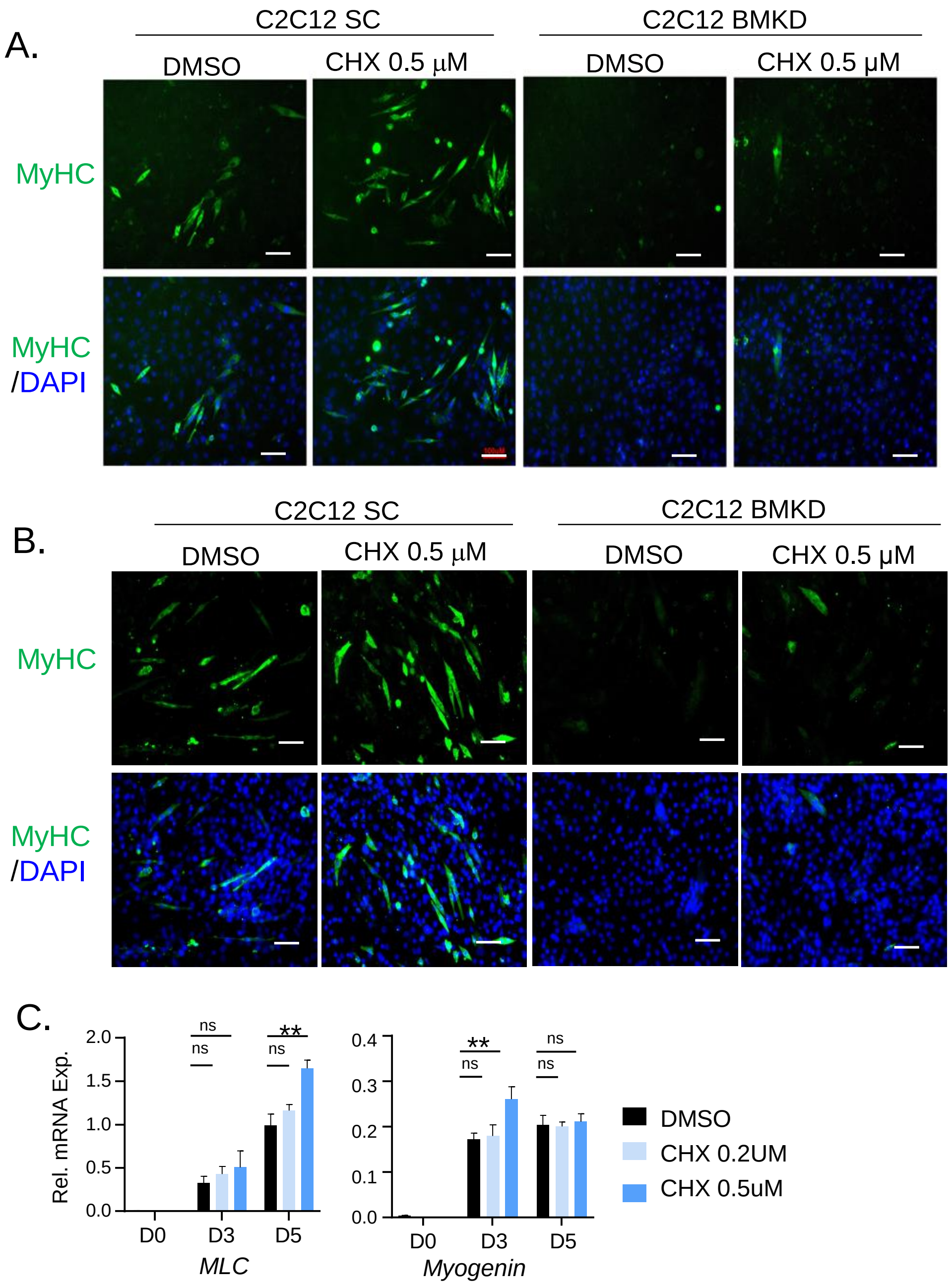

Figure S3. Effect of Chlorhexidine on C2C12 myoblast differentiation. (A, B) Representative images of immunofluorescence staining of myosin heavy chain (MyHC) at day 3 (A) and day 5 (B) of SC control and BMKD C2C12 myoblast differentiation at indicated CHX concentration. Scale bar: 100 μm. (C) RT-qPCR analysis of myogenic gene expression after CHX treatment for 24 hours at day 0, 3 and 5 of C2C12 myogenic differentiation. *MLC*: Myosin light chain. *,**: p<0.05 and 0.01 CHX vs. DMSO by Student's t test. N=3/group.

Supplemental Figure S4.

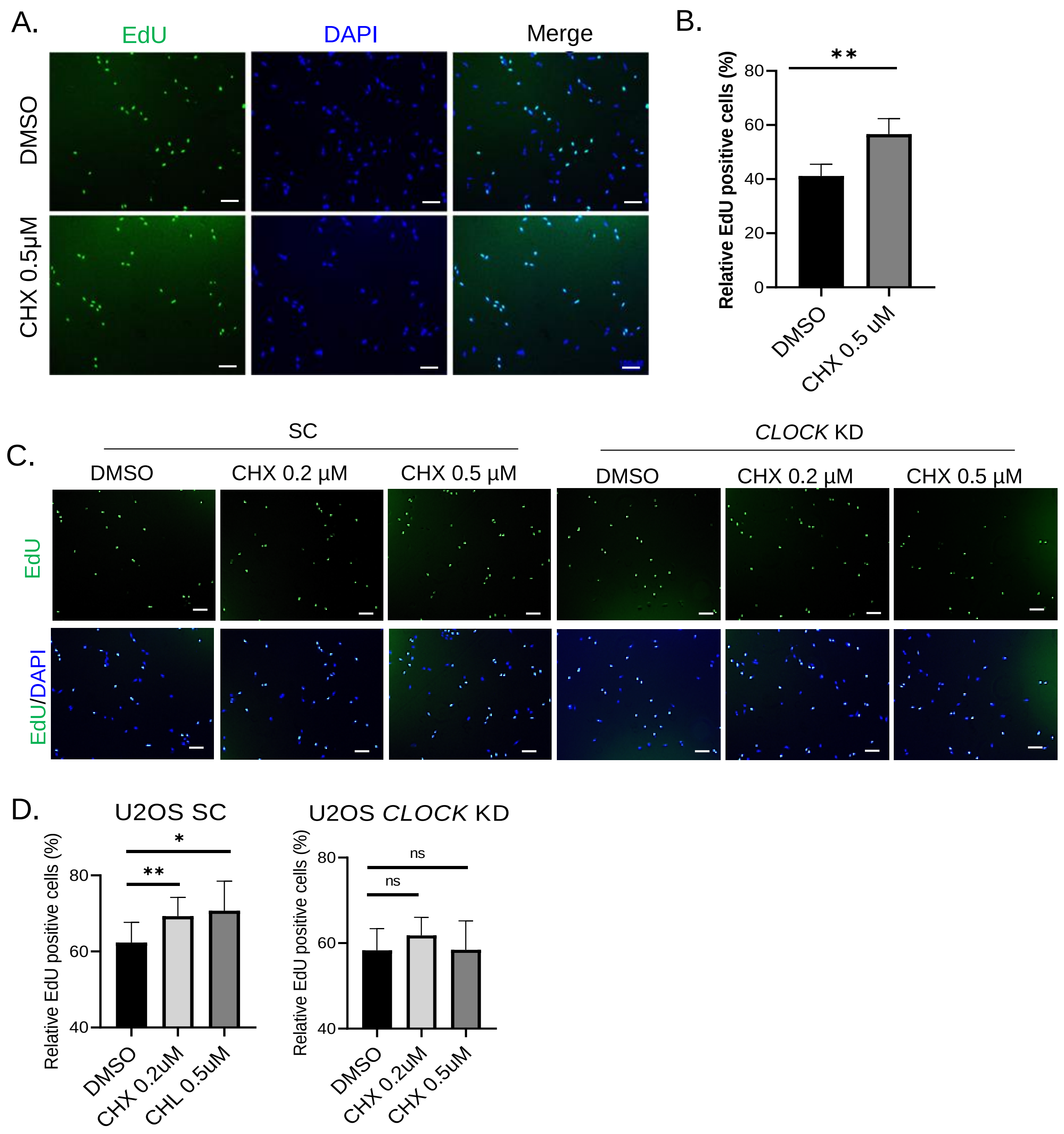

Figure S4: Effect of Chlorhexidien on proliferation of U2OS cells. (A, B) Representative images of EdU and DAPI staining of U2OS cells with DMSO or CHX treatment for 24 hours (A), with quantification (B). 3 repeats of 10 representative fields was used for quantitative analysis.. (C, D) Representative images of EdU and DAPI staining of U2OS with stable expression of scrambled control shRNA (SC) or *CLOCK* shRNA (*CLOCK* KD) treated with DMSO or CHX treatment for 24 hours (A), with quantification (B). 3 repeats of 10 representative fields was used for quantitative analysis. *,**: p<0.05 and 0.01, CHX vs. DMSO by Student's t test. Scale bar: 100 μm.

Supplemental Figure S5: Uncropped gels as for Figure 2E & 2F

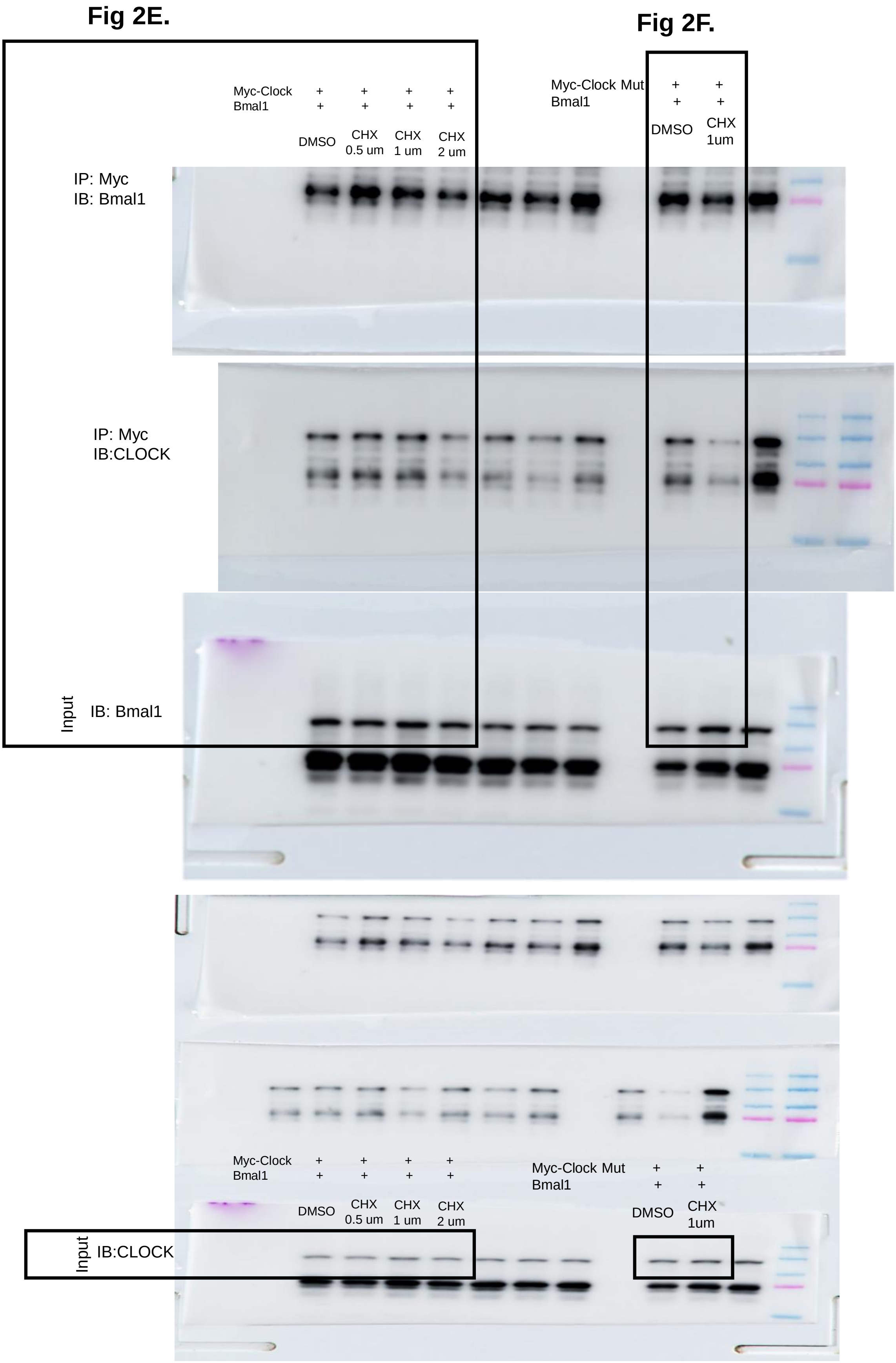

Supplemental Figure S6: Uncropped gels as for Figure 3A.

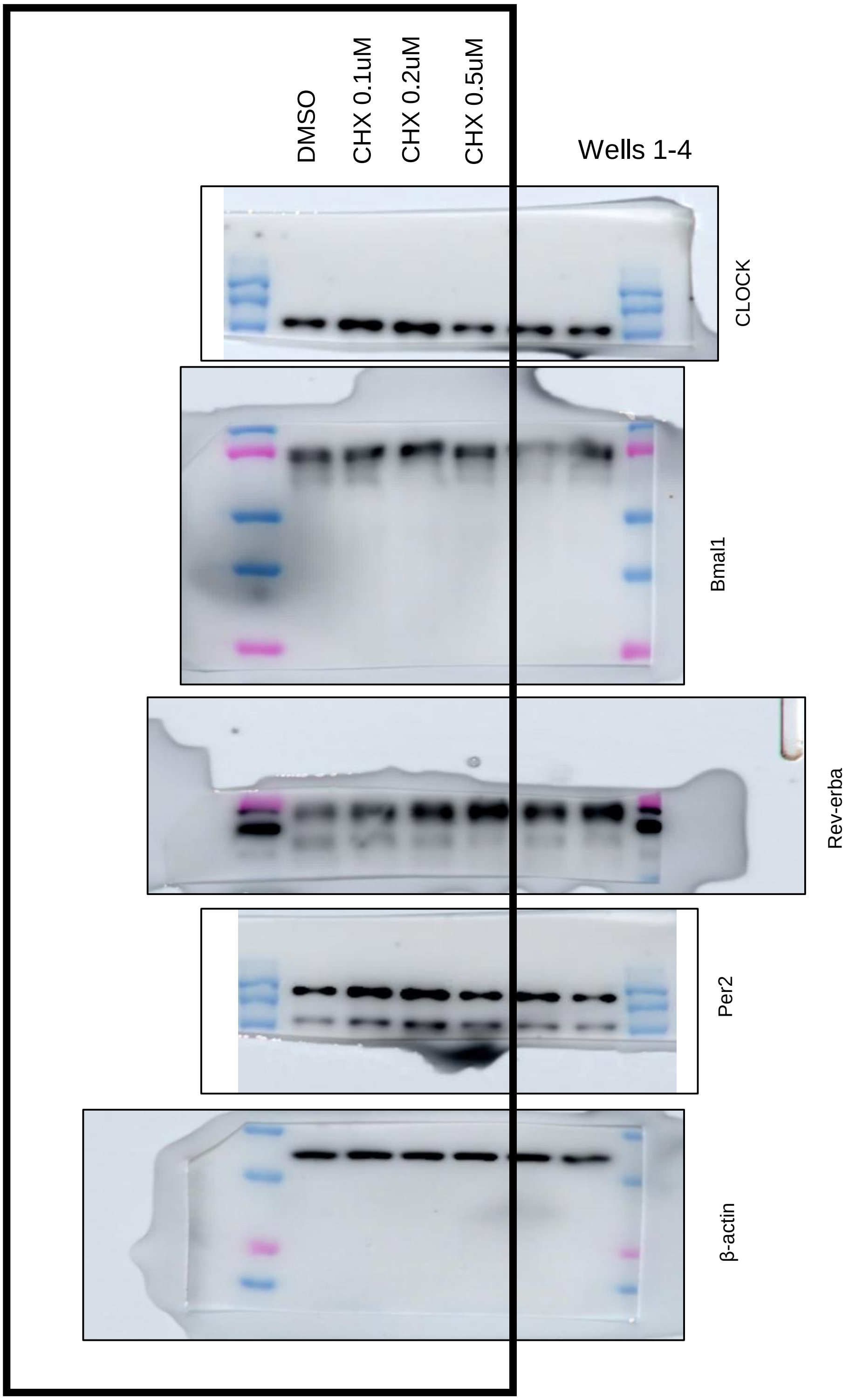

Supplemental Figure S7: Uncropped gels for Figure 4A

.

D2 wells 2-4
D4 wells 6-8
D6 wells 10-12

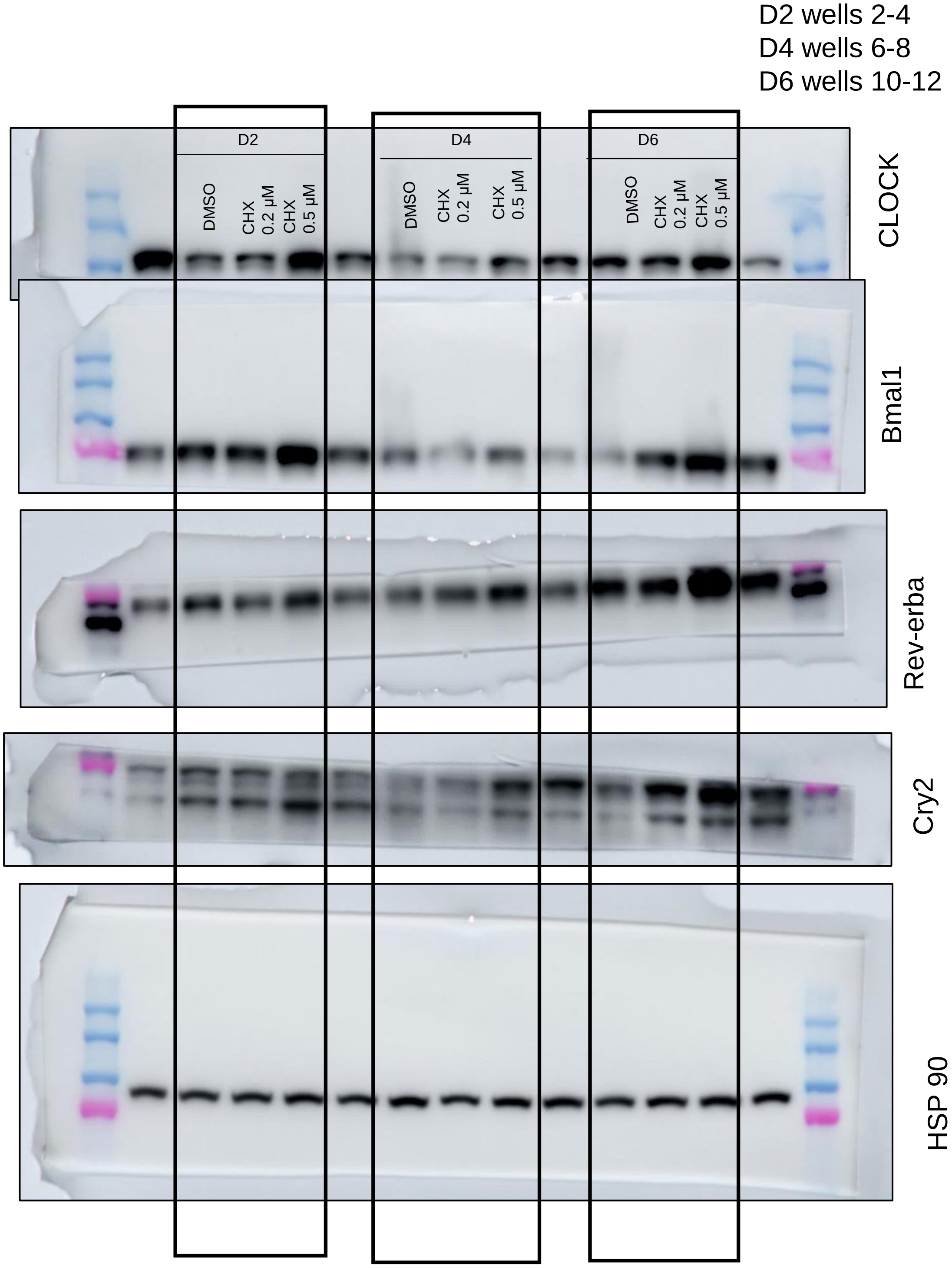

Supplemental Figure S8: Uncropped gels for Figure 5E.

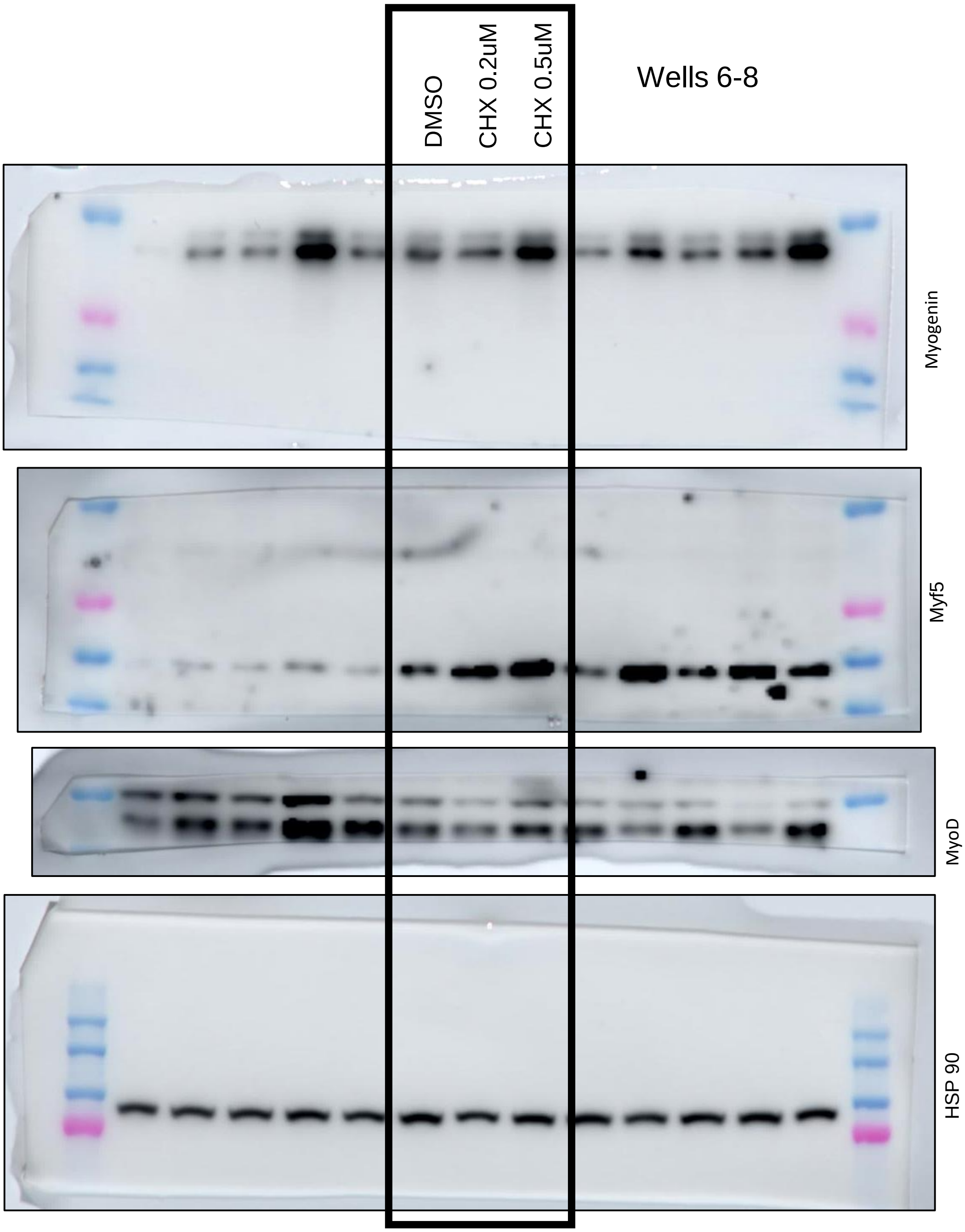

Suppl. Table 1. List of cell lines.

Cell line	Tissue	Cell type	Mediu m	Source	Obs.
**U2OS**	Bone	Epithelial	DMEM	Dr. Steve Kay at University of Southern California	Per2-Luciferase reporter<br>Liu et al. PLoS Genet. 2008 Feb 29;4(2):e1000023.
**293A**		Epithelial	DMEM	ATCC® CRL-1573	
**C2C12**	Muscle	Myoblast	DMEM	ATCC® CRL-1772	
**C2C12<br>SC control<br>Bmal1 KD**	Muscle	Myoblast	DMEM	Dr. Ke Ma at City of Hope	Bmal1 small hairpin RNA (shRNA) construct in pGIPZ vector (VGM5520–99941526, VGM5520–99342254, VGM5520–99211363) from Open Biosystems<br>Chatterjee et al. J Cell Sci 2013 May 15;126(Pt 10):2213-24.

Suppl. Table 2. List of primer sequences for RT-qPCR analysis.

Primer	Organism	Sequence
**36B4-F**	Human	AACATGCTCAACATCTCCCC
**36B4-R**	Human	CCGACTCCTCCGACTCTTC
**Bmal1-F**	Human	CGGAGTCGATGGTTCAGTTT
**Bmal1-R**	Human	CTTCCAGGACGTTGGCTAAA
**Clock-F**	Human	TGCGAGGAACAATAGACCCAA
**Clock-R**	Human	ATGGCCTATGTGTGCGTTGTA
**DBP-F**	Human	CTGATCTTGCCCTATCAAGCATT
**DBP-R**	Human	CGATGTCTTCGAGGGTCAAAG
**Per1-F**	Human	CCAGGATGTGGGAGTCTTCTAT
**Per1-R**	Human	ACGGACTTCTCCTGGGTAAA
**Per2-F**	Human	TGCAGTGGAGCAGATTCTTT
**Per2-R**	Human	GGTGGTAGCGGATTTCATTCT
**Cry1-F**	Human	CCTGTGGATTAGTTGGGAAGAA
**Cry1-R**	Human	CTACAAGACAGCCACATCCAA
**Cry2-F**	Human	GGGTCCGGGTATTTGATGAG
**Cry2-R**	Human	GTGGAAGAACTGCTGGAAGA
**NR1D1-F**	Human	TGGACTCCAACAACAACACAG
**NR1D1-R**	Human	GATGGTGGGAAGTAGGTGGG
**NR1D2-F**	Human	TTTAGTGGCATGGTTCTACTGTG
**NR1D2-R**	Human	AGCCTTCGCAAGCATGAACT
**36B4-F**	Mouse	CGCTTTCTGGAGGGTGTCCGC
**36B4-R**	Mouse	TGCCAGGACGCGCTTGTACC
**Bmal1-F**	Mouse	ACTCACCGTGCTAAGGATGGCTGT
**Bmal1-R**	Mouse	CTCGGAGACAAAGAGGATCTTCCCTCG
**Clock-F**	Mouse	TTGCTCCACGGGAATCCTT
**Clock-R**	Mouse	GGAGGGAAAGTGCTCTGTTGTAG
**DBP-F**	Mouse	CCACCGCGCAGGCTTGACAT
**DBP-R**	Mouse	ACAGGGCGAGATCAGCGGGA
**Per1-F**	Mouse	CTGCCATGGAGGAAGAAGAG
**Per1-R**	Mouse	AGCTGGGGCAGTTTCCTATT
**Per2-F**	Mouse	ATGCTCGCCATCCACAAGA
**Per2-R**	Mouse	GCGGAATCGAATGGGAGAAT
**Cry1-F**	Mouse	CTGGCGTGGAAGTCATCGT
**Cry1-R**	Mouse	CTGTCCGCCATTGAGTTCTATG
**Cry2-F**	Mouse	TGTCCCTTCCTGTGTGGAAGA
**Cry2-R**	Mouse	GCTCCCAGCTTGGCTTGA
**NR1D1-F**	Mouse	TGGCATCCGGTGCACTGCAG
**NR1D1-R**	Mouse	CCCTCCAGAAGGGTAGCACGCT
**NR1D2-F**	Mouse	GGAGTTCATGCTTGTGAAGGCTGT
**NR1D2-R**	Mouse	CAGACACTTCTTAAAGCGGCACTG
**eMyHC-F**	Mouse	AAAAGGCCATCACTGACGC
**eMyHC-R**	Mouse	CAGCTCTCTGATCCGTGTCTC
**MLC-F**	Mouse	GCAACAGGAGGACTTCAAGGAGGC
**MLC-R**	Mouse	ATTGGTGCCCAGAGCCCGGA
**Myf5-F**	Mouse	AGCTGCTGAGGGAACAGGTGGA
**Myf5-R**	Mouse	ATTCAGGCATGCCGTCAGAGCA
**MyoD-F**	Mouse	AACCCAGACCCCGTCTCCCG
**MyoD-R**	Mouse	GGGCGTGAAGAACCAGGGGC
**Myogenin-F**	Mouse	GTCCCAACCCAGGAGATCATT
**Myogenin-R**	Mouse	AGTTGGGCATGGTTTCGTCT

Suppl. Table 3. List of primary and secondary antibodies for immunoblot analysis.

Protein	Manufacturer	Catalog #	Dilution (fold)
**βActin**	Invitrogen	MA5-15739	1:10000
**HSP90**	Cell Signaling Technology	4847S	1:5000
**Bmal1**	Cell Signaling Technology	14020S	1:5000
**CLOCK**	Cell Signaling Technology	5157S	1:4000
**NR1D1**	Proteintech	14506-1-AP	1:3000
**Cry2**	Proteintech	13997-1-AP	1:4000
**Per2**	Proteintech	67513-1-Ig	1:4000
**MyoD**	Santa Cruz Biotechnology	sc377460	1:2000
**Myogenin**	Santa Cruz Biotechnology	sc52903	1:2000
**Myf5**	Santa Cruz Biotechnology	Sc518039	1:2000
**Myc**	Cell Signaling Technology	2278	1:5000
**PAX7**	DSHB	-	1:500
**Goat anti Rabbit**	Bio Rad	5213-2504	1:5000
**Goat anti Mouse**	Bio Rad	STAR 207	1:5000

Suppl. Table 4. List of primary and secondary antibodies for immunofluorescence staining.

Protein	Manufacturer	Catalog #	Dilution (fold)
**MyHC**	Developmental Studies Hybridoma Bank	MF 20	1:50
**Alexa Fluor 488 goat anti-mouse IgG**	Thermo Fisher Scientific	A-11001	1:1000
**Alexa Fluor 594 goat anti-mouse IgG**	Thermo Fisher Scientific	A-11005	1:1000
